# Patient Median-Based Quality Control in Lamotrigine Therapeutic Drug Monitoring: A 15-Year Retrospective Study

**DOI:** 10.3390/pharmaceutics18020236

**Published:** 2026-02-12

**Authors:** Anders Larsson, Mats B. Eriksson, Linda Steinholtz, Anna-Karin Hamberg

**Affiliations:** 1 Department of Medical Sciences, Clinical Chemistry and Pharmacology, Uppsala University, SE-751 85 Uppsala, Sweden; linda.steinholtz@akademiska.se (L.S.); anna-karin.hamberg@akademiska.se (A.-K.H.); 2 Department of Surgical Sciences, Uppsala University, Uppsala University Hospital, SE-751 85 Uppsala, Sweden; mats.b.eriksson@uu.se; 3 NOVA Medical School, New University of Lisbon, 1099-085 Lisbon, Portugal

**Keywords:** lamotrigine, patient median, therapeutic drug monitoring, immunosuppressive, transplantation, patient-based quality control (PBQC), pharmacokinetics, clinical laboratory quality assurance

## Abstract

**Background/Objectives:** Lamotrigine is an anticonvulsant and mood stabilizer with wide interindividual pharmacokinetic variability, necessitating therapeutic drug monitoring (TDM). Patient-based quality control (PBQC) strategies, such as tracking median drug concentrations, may complement traditional quality assurance in routine laboratory practice. **Methods:** We retrospectively analyzed 15,963 lamotrigine results collected between February 2011 and December 2025 at Uppsala University Hospital, Uppsala. Data included age, sex, sampling date, and lamotrigine concentrations. Assays were performed using the Architect platform from February 2011 to January 2021, after which the Cobas Pro c 503 platform was implemented. Yearly patient medians were calculated, and trends, seasonal variation, and method agreement were assessed. **Results:** Of all the results, 5967 were from males and 9996 from females. Median concentrations were slightly higher in males (15.20 µmol/L) than in females (13.71 µmol/L), representing a weak but statistically significant difference (Spearman R = −0.048; *p* < 0.0001). The total number of reported results increased steadily over time, from 402 in 2011 to more than 1500 annually by 2024–2025. Median lamotrigine concentrations increased from 11.85 µmol/L in 2011 to 17.40 µmol/L in 2025 (Spearman R = 0.047; *p* < 0.0001). Seasonal variation in sample volume was observed, with peaks in November and troughs in July and December, but median concentrations remained stable (CV = 3.49%). Method comparison showed strong agreement between Architect and Cobas assays (R^2^ = 0.97). **Conclusions:** Patient median lamotrigine concentrations serve as a robust PBQC tool, capable of detecting subtle analytical shifts while remaining resilient to seasonal fluctuations and platform transitions. This approach enhances confidence in assay reliability and supports safer therapeutic decision-making in real-world TDM practice.

## 1. Introduction

Lamotrigine is a widely used anticonvulsant and mood stabilizer, primarily prescribed for epilepsy and the long-term management of bipolar disorder [1,2]. Its strength lies in preventing depressive relapse, and its generally favorable tolerability profile makes it an attractive therapeutic option. Nonetheless, careful dose titration is essential to minimize the risk of severe rash, a rare but serious adverse effect [3,4].

Therapeutic drug monitoring (TDM) plays a central role in optimizing lamotrigine therapy [5,6,7]. Target plasma concentrations typically range between 5 and 60 µmol/L, balancing efficacy in seizure control with the avoidance of toxicity. The substantial interindividual variability in lamotrigine pharmacokinetics—driven by factors such as hepatic function, age, pregnancy, and concomitant enzyme-inducing or -inhibiting medications—necessitates TDM and individualized dose adjustments [8,9,10]). Recent pharmacokinetic modeling and simulation studies highlight how patient-specific characteristics can markedly influence exposure, underscoring the need for robust and adaptable measurement strategies in clinical laboratories. Retrospective real-world TDM data further illustrate the complexity of dose–concentration relationships, particularly in heterogeneous epilepsy populations undergoing polytherapy, reinforcing the importance of reliable longitudinal assay performance [11].

Traditional quality assurance in TDM relies on internal quality control and external proficiency testing. While indispensable, these approaches may not fully capture subtle, practice-level shifts that accumulate over time. Patient-based quality control (PBQC) strategies—especially those leveraging aggregated patient statistics—offer a complementary framework for continuous surveillance [12,13]. For lamotrigine, PBQC is particularly advantageous because specimens are often collected in stable clinical rhythms and across well-defined care pathways. This consistency can be harnessed to detect analytical drift in near real time, without additional costs or workflow disruption.

Tracking patient median concentrations over defined intervals represents a pragmatic PBQC approach [14,15]. Medians are inherently robust to outliers and skewed distributions, making them well suited to identify small biases or shifts that might elude conventional controls. In lamotrigine TDM, monitoring patient medians across time windows and stratified cohorts (e.g., monotherapy vs. polytherapy, plasma vs. saliva) can provide early warning signals of drift, bias, or imprecision. Such surveillance strengthens confidence in clinical interpretation and supports safer, more effective dose adjustments.

The theoretical concepts introduced—lamotrigine pharmacokinetics, the role of TDM, and the principles of patient-based quality control—are directly tied to the rationale for the study and to the interpretation of the results. Traditional quality assurance strategies, while essential, may not adequately detect gradual or practice-level drifts that can accumulate in high-throughput TDM settings.

Patient-based quality control (PBQC) provides a theoretical framework for addressing this gap. Because lamotrigine samples are often collected within relatively consistent clinical pathways, aggregated patient data—particularly median concentrations—can serve as a stable, real-world indicator of analytical performance. Medians are robust to skewed distributions and outliers, making them suitable for the early detection of systematic bias, instrument drift, or calibration errors. Thus, the theoretical premise directly informs the methodological approach of the present study: by analyzing patient median lamotrigine concentrations over time, we assess whether this PBQC strategy can enhance routine assay surveillance and complement existing quality control procedures.

In this study, we evaluate the use of patient median values as a tool for monitoring the analytical performance of lamotrigine assays in routine TDM practice.

## 2. Materials and Methods

### 2.1. Samples

Blood samples submitted for routine lamotrigine monitoring at the Departments of Clinical Chemistry and Pharmacology, Uppsala University Hospital, were collected in serum tubes (BD Vacutainer Systems, Plymouth, UK). The study period spanned from February 2011 through 1 December 2025, comprising 15,963 samples. Data extraction was performed in accordance with ethical approval, ensuring patient anonymity. Only sampling date, age (in years), sex, and lamotrigine concentrations were retained. Ethical clearance was granted by the Uppsala University Ethics Committee (Dnr 01-367).

### 2.2. Instruments

In February 2011 the Lamotrigine method was transferred to the Architect platform (Abbott Laboratories, Abbott Park, IL, USA), Uppsala, using QMS^®^ Lamotrigine reagent no. 0373795 (Microgenics, Fremont, CA, USA) together with calibrator no. 0373787 and control no. 0374090 (Microgenics). The overall coefficient of variation (CV) was 3.6% at 7.4 µmol/L and 6.0% at 54.4 µmol/L.

In January 2021, the assay was transferred to the Cobas Pro c 503 platform (Roche Diagnostics, Rotkreuz, Switzerland), employing ARK Lamotrigine reagent no. 5023-0001-00 (ARK Diagnostics, Fremont, CA, USA) along with ARK calibrators and controls. External quality controls were provided monthly by LGC Standards (Wesel, Germany). The CV was 5.3% at 7.3 µmol/L and 4.7% at 45 µmol/L. The therapeutic reference range was 5–60 µmol/L.

### 2.3. Statistical Calculations

Yearly median lamotrigine values were calculated. Statistical evaluations were conducted using Excel 365 (Microsoft Corp., Seattle, WA, USA) and Statistica 10 (Tibco Software, Palo Alto, CA, USA). A non-parametric comparison of lamotrigine concentrations between males and females was performed using the Mann–Whitney U test. The null hypothesis (H_0_) stated that the distributions did not differ by sex.

## 3. Results

### 3.1. Number of Lamotrigine Results and Sex Distribution

**Figure 1:**
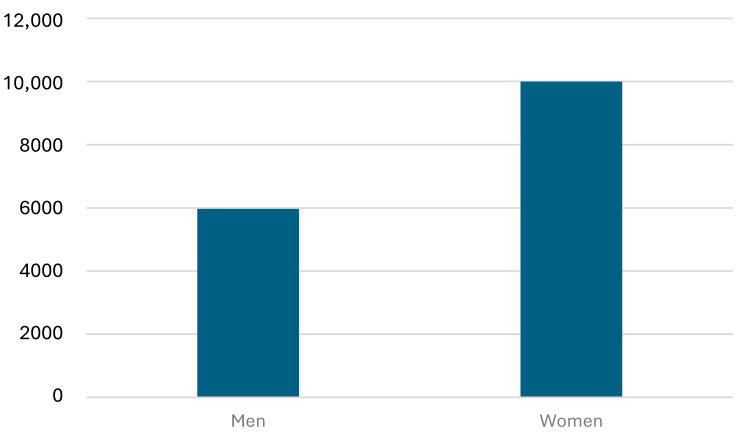
Number of lamotrigine analyses stratified by sex.

Between 2011 and 2025 a total of 15,963 lamotrigine results were reported. Of these, 6164 were from males and 10331 from females (χ^2^ = 534.8559; *p* < 0.0001) (Figure 1).

The median lamotrigine concentration was 13.71 µmol/L in females and 15.20 µmol/L in males (Table 1). This difference was highly significant according to the Mann–Whitney U test (Z = 6.162; *p*-value 7.2 × 10^−11^). The number of lamotrigine results below 10 µg/L was 5818 and 8450 results were below 15 µg/L, thus below the therapeutic interval. On the other hand 5385 results were above 20 µg/L and 3863 results were above 25 µg/L.

The simulated medians closely matched the reported values, supporting the use of the fitted distributions for this purpose. Under the assumptions of the log normal approximation, these results suggest that the underlying distributions differ between sexes, with males having higher values than females.

### 3.2. Changes in the Number of Reported Lamotrigine Results over Time

**Figure 2:**
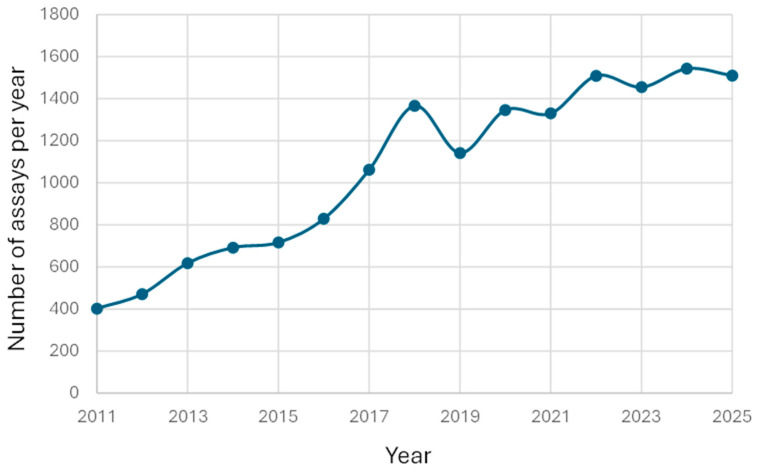
Number of lamotrigine results/year. The data is presented for the time period 1 February 2011–1 December 2025.

The number of reported results increased from 402 in 2011 to 1543 in 2024 (Figure 2). During the first eleven months of 2025 the number of reported results were 1510, indicating that the total for 2025 will exceed the value for 2024.

### 3.3. Changes in Median Lamotrigine Concentrations over Time

**Figure 3:**
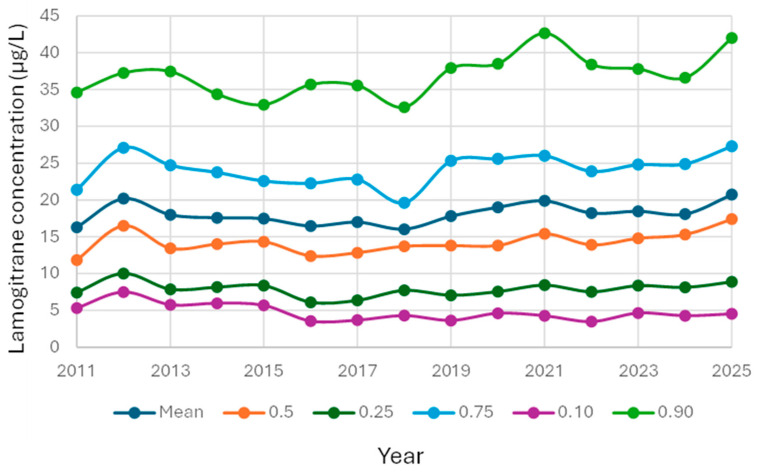
Lamotrigine results over time. The data is presented as 10th percentile, lower quartile, mean, median, upper quartile and 90th percentile per year for the time period 1 February 2011–1 December 2025.

The median lamotrigine concentrations increased over time from 11.85 µmol/L in 2011 to 17.40 µmol/L in 2025 (Figure 3). This trend represented a weak but highly significant change according to Spearman’s rank test (R = 0.047; *p* < 1 × 10^−9^).

### 3.4. Seasonal Variation in Lamotrigine Results

**Figure 4:**
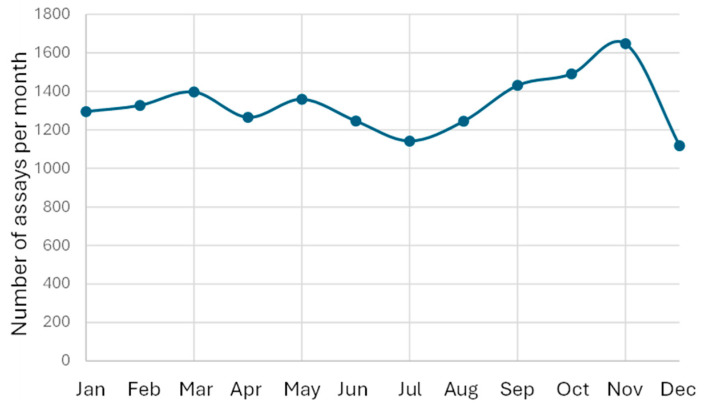
Number of lamotrigine results/month. The data is presented for the time period 2011–1 December 2025.

**Figure 5:**
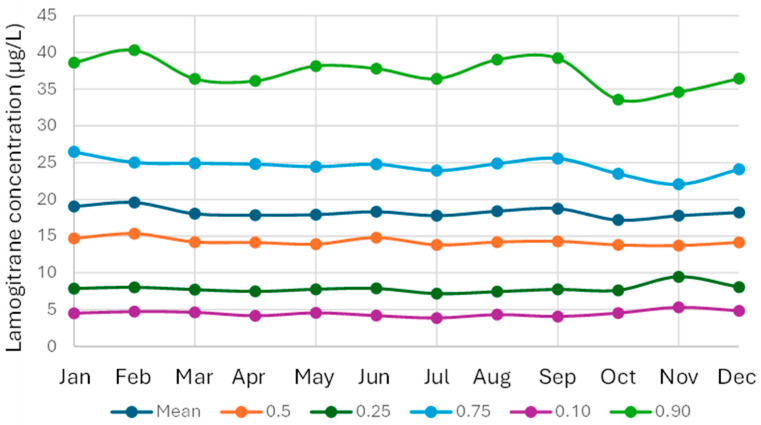
Lamotrigine results per month. The data is presented as 10th percentile, lower quartile, mean, median, upper quartile, and 90th percentile per year for the time period 1 February 2011–1 December 2025.

The number of reported lamotrigine results was highest in November and lowest in July and December (Figure 4). July is the main vacation month in Sweden and December is also a holiday month. Despite these variations in the number of reported test results across months, the median lamotrigine concentrations remained stable, with a coefficient of variation for the monthly medians of 3.49% (Figure 5).

### 3.5. Method Comparison Between the Architect and Cobas Lamotrigine Methods

**Figure 6:**
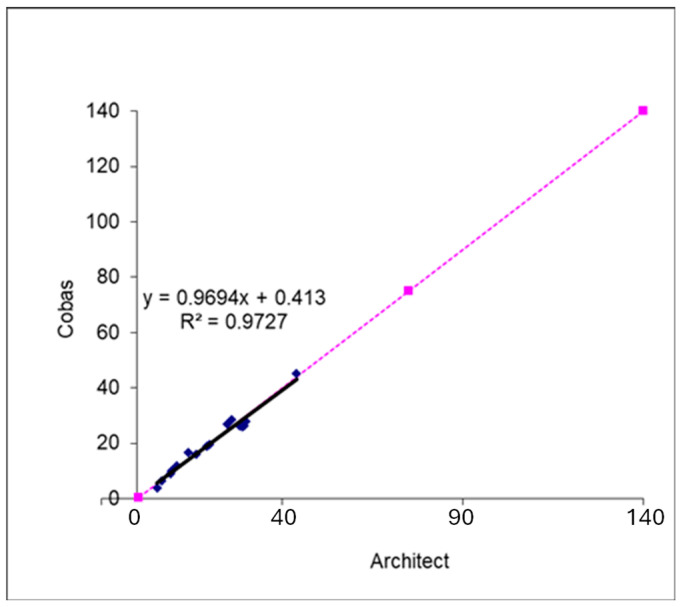
Comparison between the Architect and Cobas lamotrigine methods using patient samples. The blue dots represent individual patient sample results measured on the two analytical platforms. The purple dashed line with squares represents the line of identity (45-degree line), illustrating where results would fall if the two methods produced identical values. The black regression line shows the observed relationship, with the displayed equation and R^2^ value indicating the strength of the correlation.

The comparison between the Architect and Cobas lamotrigine methods during the validation process of the Cobas method showed good agreement for patient samples (y = 0.97x + 0.413; R^2^ = 0.97) (Figure 6).

## 4. Discussion

This large-scale retrospective study demonstrates the value of using patient median values as a pragmatic PBQC strategy for lamotrigine monitoring. Over a 14-year period, we observed consistent trends in assay performance, with increasing median concentrations and rising test volumes reflecting both clinical uptake and evolving therapeutic practice. The weak but statistically significant sex-related differences in median concentrations are consistent with known pharmacokinetic variability, although the magnitude of these differences is unlikely to justify sex-specific reference ranges.

Importantly, seasonal fluctuations in sample volume did not lead to instability in median values, underscoring the robustness of patient medians as a quality control metric. This stability highlights their suitability for continuous monitoring, even in healthcare systems that experience predictable variations in patient flow due to holidays or staffing cycles.

The transition from the Architect to the Cobas platform in 2021 provided a natural opportunity to assess PBQC applicability. The strong agreement between methods (R^2^ = 0.97) confirms analytical continuity, and patient medians served as an independent safeguard against unnoticed drift during implementation. This real-world validation illustrates how PBQC can complement conventional internal and external quality controls, which may miss subtle practice-level shifts.

The gradual increase in median lamotrigine concentrations over time may reflect evolving prescribing patterns, wider adoption of TDM, or shifts in patient demographics. Although the statistical correlation was weak, its significance suggests that longitudinal monitoring of medians can reveal subtle population-level trends that merit further clinical investigation.

Together, these findings support the integration of patient median tracking into routine lamotrigine TDM workflows. By leveraging data already generated during clinical care, laboratories can enhance quality surveillance without additional cost or workload. This approach not only strengthens analytical reliability but also increases confidence in therapeutic decision-making, ultimately promoting patient safety.

Median plasma lamotrigine concentrations were slightly more than 10% higher in males than in females. This difference should be interpreted cautiously, because the analysis is based on approximate distributions derived from summary statistics rather than individual-level data. Nonetheless, the very small *p*-value from the simulated Mann–Whitney U test indicates that the null hypothesis of identical distributions between males and females can be rejected.

Lamotrigine is metabolized primarily in the liver through UDP-glucuronosyltransferase-mediated glucuronidation, predominantly via UGT1A4, while cytochrome P450 enzymes play only a minimal and clinically negligible role in its metabolism [16,17].

Lamotrigine pharmacokinetics exhibit clinically relevant interindividual variability driven by sex, age, pregnancy, and concomitant antiepileptic and hormonal therapy, underscoring the need for therapeutic drug monitoring (TDM). This aligns with the high number of lamotrigine analyses in our study. Sex-related differences, age, and exposure to exogenous estrogens (combined oral contraceptives and hormone-replacement therapy) substantially influence lamotrigine clearance and plasma concentrations, with important implications for monitoring.

Combined oral contraceptives and other estrogen-containing preparations increase lamotrigine glucuronidation and reduce steady-state concentrations, depending on the hormonal regimen [18]. Pregnancy and rising estradiol levels markedly increase lamotrigine clearance, often necessitating dose escalation and close TDM to avoid seizure breakthrough [19]. Hormone-replacement therapy may produce similar reductions in lamotrigine concentrations in postmenopausal women [20]. While routine, universally applied TDM for lamotrigine is debated [21], targeted monitoring is supported when factors known to alter clearance are present, or when clinical response changes [20]. Evidence supports a pragmatic, individualized TDM approach for lamotrigine—prioritizing patients on polytherapy, women initiating or changing estrogenic therapies, pregnant patients, and those with age-related pharmacokinetic changes—to optimize efficacy and safety [6].

In routine PBQC implementation, defining a pragmatic threshold for when a change in the patient median should trigger investigation is essential. Several laboratories using PBQC frameworks have found that shifts of approximately 5–10% in the patient median—persisting over several days or multiple analytical batches—generally indicate meaningful analytical or preanalytical issues rather than random variation. For lamotrigine specifically, the monthly coefficient of variation around the median in this study was approximately 3%, demonstrating that the natural biological and workflow-related noise is low. Against this background, a sustained deviation exceeding ±10% from the expected median would therefore be unlikely to arise from random fluctuations and should prompt a review of calibrations, reagent lots, instrument performance, and sample handling workflows.

Smaller deviations (e.g., 5–7%) may still merit attention if they show a consistent directional trend, especially around the time of instrument maintenance, reagent lot changes, or clinical workflow disruptions. Conversely, isolated one day fluctuations are typically attributable to random patient mix effects and should be interpreted with caution.

The performance of PBQC for lamotrigine in this study compares favorably with its application to other routinely monitored therapeutic drugs, particularly immunosuppressants. Lamotrigine displays several features that enhance PBQC performance. First, its intraindividual variability is generally lower under stable dosing conditions, particularly in patients without interacting medications. Second, lamotrigine is typically prescribed for chronic conditions—epilepsy and bipolar disorder—where dose adjustments are less frequent compared with immunosuppressants, leading to more stable population distributions. Third, test volumes for lamotrigine were high and consistent across many years in this study, producing robust medians with low natural variation (monthly CV ≈ 3%). The combination of stable pharmacokinetics, large sample numbers, and relatively predictable patient mix results in exceptionally smooth long-term median curves. This stability facilitates early detection of analytical drift because even small systematic deviations become apparent against the otherwise steady background signal.

There are limitations when interpreting the findings of this study. First, the retrospective design inherently restricts control over patient level variables such as diagnosis, dosing regimens, comorbidities, adherence, and concomitant medications, all of which may influence lamotrigine concentrations. The dataset therefore reflects routine clinical practice rather than a controlled population, which limits its ability to attribute observed trends to specific clinical factors. Second, demographic information was limited to age and sex; other pharmacokinetically relevant factors—including pregnancy status, use of estrogen-containing therapies, renal or hepatic impairment, and enzyme-inducing or -inhibiting co-medications—were not available. This constrains interpretation of observed sex differences and broader temporal changes. Third, a platform transition occurred during the 14 year study period. Although method comparisons and external quality assessment demonstrated good agreement, subtle differences in calibration or matrix effects between assays may have contributed to long-term trends in patient medians. Fourth, the analysis relied on aggregated summary statistics rather than repeated measurements from the same individuals. This prevented assessment of intraindividual variability or longitudinal changes within specific patient trajectories.

This study demonstrates that patient median lamotrigine concentrations provide a robust and informative patient-based quality control metric in routine therapeutic drug monitoring. Across 14 years of data, patient medians remained stable despite substantial seasonal fluctuations in sample volume and two major analytical platform transitions. Sex-specific differences and long-term increases in median concentrations were small but detectable, illustrating the sensitivity of PBQC to subtle population level trends while retaining resilience to short term variability.

Strong analytical agreement between the Architect and Cobas platforms confirmed measurement continuity, and the close alignment of patient medians across methods further supports their use as an independent, real-world safeguard against unnoticed drift. Because medians are simple to compute, resistant to outliers, and derived from routinely generated clinical data, their implementation requires minimal additional resources.

Together, these findings support incorporating patient median monitoring into standard lamotrigine TDM workflows as a complementary tool to traditional quality control systems. By strengthening confidence in assay stability and long-term performance, PBQC has the potential to improve the reliability of therapeutic decision-making and contribute meaningfully to patient safety in everyday clinical practice.

## Figures and Tables

**Table 1:** Descriptive statistics (mean, median, selected percentiles) of the study variable for the total sample, males, and females.

	Valid N	Mean	Median	0.25	0.75	0.10	0.90
All	15,963	18.23	14.06	7.86	24.50	4.49	37.50
Males	5967	19.04	15.20	8.11	25.90	4.51	39.31
Females	9996	17.75	13.71	7.70	23.70	4.48	36.11

## Data Availability

The dataset used and analyzed during the current study is available from the corresponding author on request.

## References

[1] Besag F.M.C., Vasey M.J., Sharma A.N., Lam I.C.H. (2021). Efficacy and safety of lamotrigine in the treatment of bipolar disorder across the lifespan: A systematic review. Ther. Adv. Psychopharmacol..

[2] Prabhavalkar K.S., Poovanpallil N.B., Bhatt L.K. (2015). Management of bipolar depression with lamotrigine: An antiepileptic mood stabilizer. Front. Pharmacol..

[3] Zhang L., Yang P., Zhu Y., Liu K., Sun Z. (2025). Toxic epidermal necrolysis following lamotrigine replacement therapy in a woman planning pregnancy: A case report and literature review. BMC Women’s Health.

[4] De Bellis M., d’Orsi G., Rubino E.M., Arigliano C., Carella M., Sciruicchio V., Liantonio A., De Luca A., Imbrici P. (2025). Adverse effects of antiseizure medications: A review of the impact of pharmacogenetics and drugs interactions in clinical practice. Front. Pharmacol..

[5] Rambeck B., Wolf P. (1993). Lamotrigine clinical pharmacokinetics. Clin. Pharmacokinet..

[6] Goo Y., der Nederlanden A.M., Bleasel A., Alffenaar J.W., Kim H.Y. (2024). Dose Monitoring of Lamotrigine Monotherapy in Pregnancy: Are Pregnant Women with Epilepsy Currently Optimally Managed? A Systematic Review. Ther. Drug Monit..

[7] Li J.C., Miao C.F., Lei Y., Liu A.L. (2025). Physiologically Based Pharmacokinetic Modeling to Predict Lamotrigine Exposure in Special Populations to Facilitate Therapeutic Drug Monitoring and Guide Dosing Regimens. Pharmaceuticals.

[8] Reimers A., Skogvoll E., Sund J.K., Spigset O. (2007). Lamotrigine in children and adolescents: The impact of age on its serum concentrations and on the extent of drug interactions. Eur. J. Clin. Pharmacol..

[9] Böttiger Y., Svensson J.O., Ståhle L. (1999). Lamotrigine drug interactions in a TDM material. Ther. Drug Monit..

[10] Yamamoto Y., Usui N., Kagawa Y., Imai K. (2024). Time-Course Changes in Lamotrigine Concentration after Addition of Valproate and the Safety and Long-Term Tolerability of Lamotrigine-Valproate Combination Therapy. Biol. Pharm. Bull..

[11] Lee Z.N., van Nuland M., Bognàr T., Leijten F.S.S., van der Elst K.C.M. (2024). Association of Lamotrigine Plasma Concentrations with Efficacy and Toxicity in Patients with Epilepsy: A Retrospective Study. Ther. Drug Monit..

[12] van Rossum H.H. (2022). Technical quality assurance and quality control for medical laboratories: A review and proposal of a new concept to obtain integrated and validated QA/QC plans. Crit. Rev. Clin. Lab. Sci..

[13] Loh T.P., Cervinski M.A., Katayev A., Bietenbeck A., van Rossum H., Badrick T. (2019). Recommendations for laboratory informatics specifications needed for the application of patient-based real time quality control. Clin. Chim. Acta.

[14] Larsson A., Hamberg A.K., Cedernaes J., Hallberg P., Karlqvist J.H., Karlsson M. (2025). New Monitoring Recommendations for Digoxin During the Last Decade Are Associated with Decreased Serum Digoxin Concentrations in Patient Samples. Basic Clin. Pharmacol. Toxicol..

[15] Larsson A., Saldeen J., Duell F. (2025). Recent decline in patient serum folate test levels using Roche Diagnostics Folate III assay. Clin. Chem. Lab. Med..

[16] Elwes R.D., Binnie C.D. (1996). Clinical pharmacokinetics of newer antiepileptic drugs. Lamotrigine, vigabatrin, gabapentin and oxcarbazepine. Clin. Pharmacokinet..

[17] Gardner I., Heikkinen A.T., Tang L.W.T., Lapham K., Goosen T.C. (2025). Development of a PBPK Model for Lamotrigine which Incorporates Metabolism by UGT2B10: Impact of UGT2B10 Poor Metabolizer Phenotype and Pregnancy. AAPS J..

[18] Sabers A., Ohman I., Christensen J., Tomson T. (2003). Oral contraceptives reduce lamotrigine plasma levels. Neurology.

[19] Christensen J., Petrenaite V., Atterman J., Sidenius P., Ohman I., Tomson T., Sabers A. (2007). Oral contraceptives induce lamotrigine metabolism: Evidence from a double-blind, placebo-controlled trial. Epilepsia.

[20] Reimers A. (2017). Hormone replacement therapy with estrogens may reduce lamotrigine serum concentrations: A matched case-control study. Epilepsia.

[21] Chong E., Dupuis L.L. (2002). Therapeutic drug monitoring of lamotrigine. Ann. Pharmacother..

